# Comparative evaluation of the impact on endothelial cells induced by different nanoparticle structures and functionalization

**DOI:** 10.3762/bjnano.6.28

**Published:** 2015-01-27

**Authors:** Lisa Landgraf, Ines Müller, Peter Ernst, Miriam Schäfer, Christina Rosman, Isabel Schick, Oskar Köhler, Hartmut Oehring, Vladimir V Breus, Thomas Basché, Carsten Sönnichsen, Wolfgang Tremel, Ingrid Hilger

**Affiliations:** 1 Institut für Diagnostische und Interventionelle Radiologie des Klinikums der Friedrich-Schiller-Universität Jena, Bachstraße 18, D-07740 Jena, Germany; 2 Institut für Diagnostische und Interventionelle Radiologie des Klinikums der Friedrich-Schiller-Universität Jena, Forschungszentrum Lobeda, Erlanger Allee 111, D-07747 Jena, Germany; 3 Institut für Physikalische Chemie, Johannes Gutenberg Universität Mainz, Duesbergweg 10–14, D-55128 Mainz, Germany; 4 Johannes Gutenberg-Universität, Institut für Anorganische Chemie und Analytische Chemie, Duesbergweg 10–14, D-55128 Mainz, Germany; 5 Institut für Anatomie II, Universitätsklinikum Jena, Teichgraben 7, D-07743 Jena, Germany

**Keywords:** cell viability, gold nanoparticles, internalization, Janus particles, quantum dots

## Abstract

In the research field of nanoparticles, many studies demonstrated a high impact of the shape, size and surface charge, which is determined by the functionalization, of nanoparticles on cell viability and internalization into cells. This work focused on the comparison of three different nanoparticle types to give a better insight into general rules determining the biocompatibility of gold, Janus and semiconductor (quantum dot) nanoparticles. Endothelial cells were subject of this study, since blood is the first barrier after intravenous nanoparticle application. In particular, stronger effects on the viability of endothelial cells were found for nanoparticles with an elongated shape in comparison to spherical ones. Furthermore, a positively charged nanoparticle surface (NH_2_, CyA) leads to the strongest reduction in cell viability, whereas neutral and negatively charged nanoparticles are highly biocompatible to endothelial cells. These findings are attributed to a rapid internalization of the NH_2_-functionalized nanoparticles in combination with the damage of intracellular membranes. Interestingly, the endocytotic pathway seems to be a size-dependent process whereas nanoparticles with a size of 20 nm are internalized by caveolae-mediated endocytosis and nanoparticles with a size of 40 nm are taken up by clathrin-mediated internalization and macropinocytosis. Our results can be summarized to formulate five general rules, which are further specified in the text and which determine the biocompatibility of nanoparticles on endothelial cells. Our findings will help to design new nanoparticles with optimized properties concerning biocompatibility and uptake behavior with respect to the respective intended application.

## Introduction

To advance the field of nanomedicine, innovative nanoparticle formulations with suitable properties for diagnostic imaging, therapy (e.g., magnetic hyperthermia), delivery of drugs and siRNA have been developed. Apart from their feasibility for the respective application many of these investigations revealed that the most important factors affecting cell viability and internalization by human cells are the type of metal (e.g., inorganic noble metal, metal oxide, semiconductor nanoparticles), the shape (e.g., rods, spheres, asymmetric assemblies) and the surface charge (negative, neutral or positive).

Gold nanoparticles exhibit strong light scattering and absorption at their resonance wavelength due to their plasmonic properties [1–2]. Thus, these particles are used for optical imaging approaches [3–4]. Moreover, applications as contrast media for CT [5–6] and for selective cell targeting [7] are suggested. Gold nanorods were shown to have better optical imaging properties compared to spherical gold nanoparticles [8–10]. Importantly, the cytotoxicity of gold nanoparticles depends on the surface coating. Cetyltrimethylammonium bromide (CTAB), an important material during synthesis, was cytotoxic to many cell lines [11–14], rendering an appropriate coating of gold nanoparticles indispensable for biocompatibility.

Metal oxide based nanoparticles such as iron oxide and manganese oxide are ideal tools for MRI applications. They are easy to synthesize and they showed excellent magnetization curves leading to *T*2 and *T*1 relaxivities during MRI [15–20]. Owing to their magnetic properties, they can particularly be used for hyperthermia applications and magnetic targeting through the body [21–27]. An assembly of multiple nanoparticles to form double-sided asymmetric shapes right up to nanoflowers offers the possibility for multimodal imaging and multiple drug loading without steric hindrance [28–31]. These nanoparticles are very new in the field of nanomedicine and poorly investigated despite their interesting features.

Colloidal semiconductor nanocrystals or quantum dots (QDs) with their outstanding fluorescence properties also play a distinct role in life science. QDs exhibit high stability against photo bleaching and they are easily tunable in color. Several studies demonstrated their feasibility for detection of molecular markers through optical imaging technologies [32–35]. For therapeutic purposes, the use of QDs was hypothesized to induce a localized inactivation of tumor cells after radiofrequency field irradiation [36].

Nearly all biomedical applications implicate intravenous application of nanoparticle. Nanoparticles will finally enter the blood system and exhibit specific interactions with the corresponding cellular and serum components. Interactions of nanoparticles with cells will be distinctly influenced by the properties of the respective nanoparticle formulation, caused by its size, shape and nature of surface charge/coating [37]. In this context, a discrete size-dependency was observed, according to which larger particles seemed to be less cytotoxic than smaller ones [38–42]. In contrast, rather controversial findings have been reported in relation to the shape of gold nanoparticles. Recently, it has been shown that macrophages exhibit a higher uptake of rods than spheres [43], whereas in prostate cells the uptake of spheres was more efficient compared to PEGylated rods [44]. Analysis of epithelial cells showed no significant difference in uptake between rods and spheres [45].

The recent advantages in knowledge and the wide field of potential applications make it necessary to identify general principles of interaction between nanoparticle design (shape, surface charge, metal component) and the resulting effects on cell metabolism and internalization. Therefore, the scope of this study was directed to examine general rules of nanoparticle processing in endothelial cells. All nanoparticle formulations used in this study are listed in Table 1.

**Table 1:** The nanoparticle used in this study and their characteristics.

nanoparticle	functionalization	charge	shape	TEM image

Au	CTAB	0	spheres	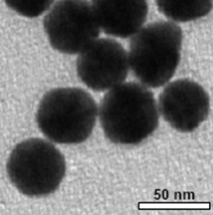
Au	PEG-OCH_3_	+/−	spheres	
Au	PEG-COOH	−	spheres	
Au	PEG-NH_2_	+/−	spheres	
Au	CTAB	0	rods	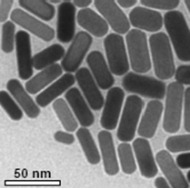
Au	PEG-OCH_3_	+/−	rods	
Au	PEG-COOH	−	rods	
Au	PEG-NH_2_	+	rods	
Au@Fe_3_O_4_	silica-PEG	−	asymmetric	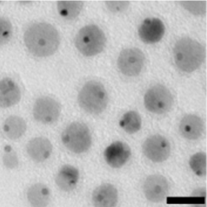
Au@Fe_3_O_4_	silica-PEG–NH_2_	+	asymmetric	
Au@MnO	silica-PEG	−	asymmetric	
Au@MnO	silica-PEG–NH_2_	+	asymmetric	
MnO	silica-PEG	−	spheres	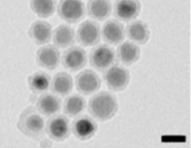
Fe_3_O_4_	silica-PEG	−	spheres	
QDs	DPA	+/−		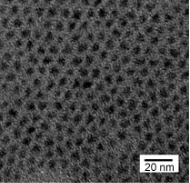
QDs	MPA	−		
QDs	CyA	+		

To investigate the impact of the nanoparticle shape on cell metabolism and internalization, we compared gold nanoparticles with rod-like and spherical shapes. Furthermore, we 1) analyzed the behavior of spherical metal oxide nanoparticles in comparison to asymmetric elongated gold@metal oxide nanoparticles, 2) determined the impact of different nanoparticle materials on cell life, 3) investigated the effects of the surface coating and the surface charge of QDs (cationic, anionic, or neutral) on cell metabolism, membrane integrity and uptake, 4) monitored the cellular localization depending on the size and shape of different nanoparticles and finally 5) investigated endocytotic pathways of nanoparticles to gather insights into their uptake mechanisms.

## Results and Discussion

### Impact of the nanoparticle shape on endothelial cells

**Figure 1:**
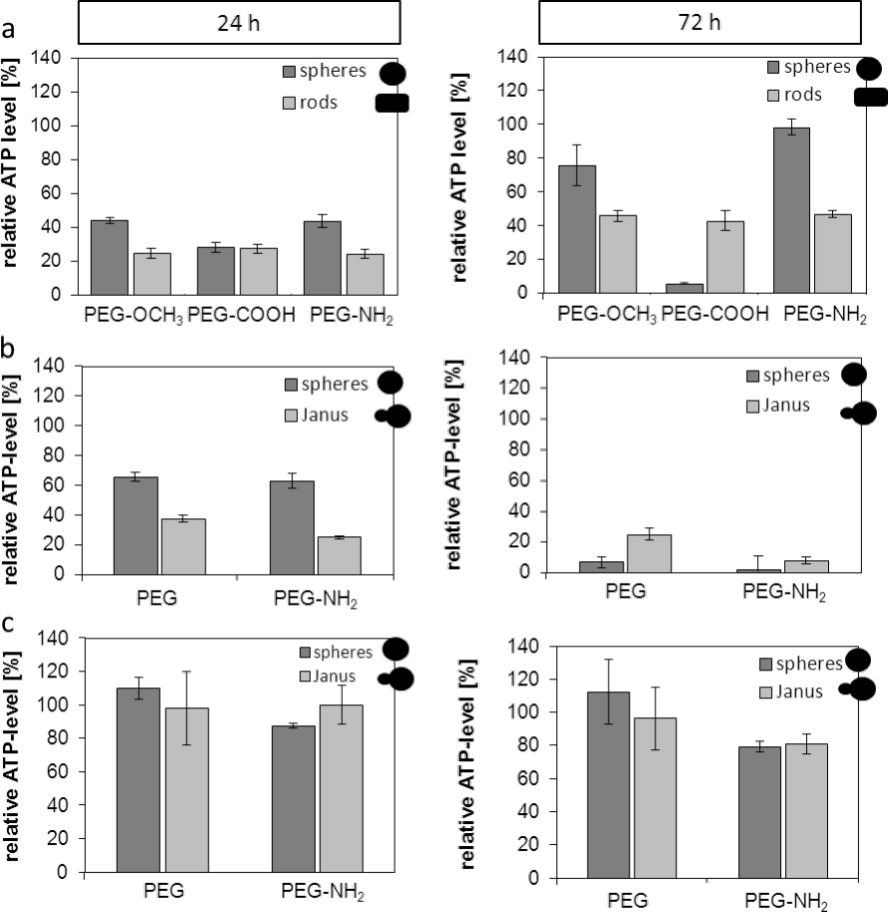
Impact of different shaped and functionalized nanoparticles on the cellular ATP-level of different endothelial cells after 24 h and 72 h of incubation. Relative cellular ATP-levels were detected by ATPLite assay. (a) SVEC4-10 were treated with 30 µg/mL of gold nanoparticles functionalized with OCH_3_, COOH or NH_2_. (b) HMEC-1 cells were treated with 20 µg/mL of MnO and Au@MnO nanoparticles. (c) HMEC-1 cells were treated with 20 µg/mL of Fe_3_O_4_ and Au@Fe_3_O_4_ nanoparticles. Data were normalized to control values (no particle exposure), which were set to 100% ATP level.

Our investigations revealed that the strongest reduction of cellular ATP levels occurred after 24 h of incubation with the OCH_3_- and NH_2_-functionalized gold rods compared to the spherical ones. After 72 h of incubation, the same trend occurred for the OCH_3_- and NH_2_-functionalized gold rods. This finding was similar to the results observed after 24 h of incubation with a distinctly high impact of the gold rods on cell metabolism. Only for the COOH-functionalized particles, we observed no differences regarding the nanoparticle shape (Figure 1a) after 24 h of incubation. Interestingly after 72 h, the spherically shaped particles had a stronger effect on cell metabolism (Figure 1a). This effect after 72 h of incubation implies first long term effects of the nanoparticles in vitro. In most of other studies investigating nanoparticle cytotoxicity only short incubation times of 5 to 24 h were demonstrated. The recovery of ATP levels after 72 h could be attributed to ATP-consuming processes, such as the uptake of nanoparticles (decreased ATP levels after 24 h of incubation) [46]. Among the nanoparticles made up of MnO and Fe_3_O_4_, again, those with an elongated shape (Au@MnO and Au@Fe_3_O_4_, Figure 1b and Figure 1c) led to a stronger reduction of ATP levels than the spherical ones in a time-dependent manner. In general, the MnO-based nanoparticles and nanoparticles with NH_2_-functionalization had a stronger impact on cell metabolism than the Fe_3_O_4_ variants or the formulations without NH_2_-functionalization. The mentioned relationships could be attributed to the positively charged surface (NH_2_), which has been reported to induce damages on the cell membranes (APTMS-coated nanoparticles [47]). To conclude, among the noble metal and metal oxide nanoparticles the shape seems to have a higher influence on cell metabolism than the surface coating and the resulting charge.

### The number of ZnS monolayers (ML) and the surface coating of QDs influences cell viability

**Figure 2:**
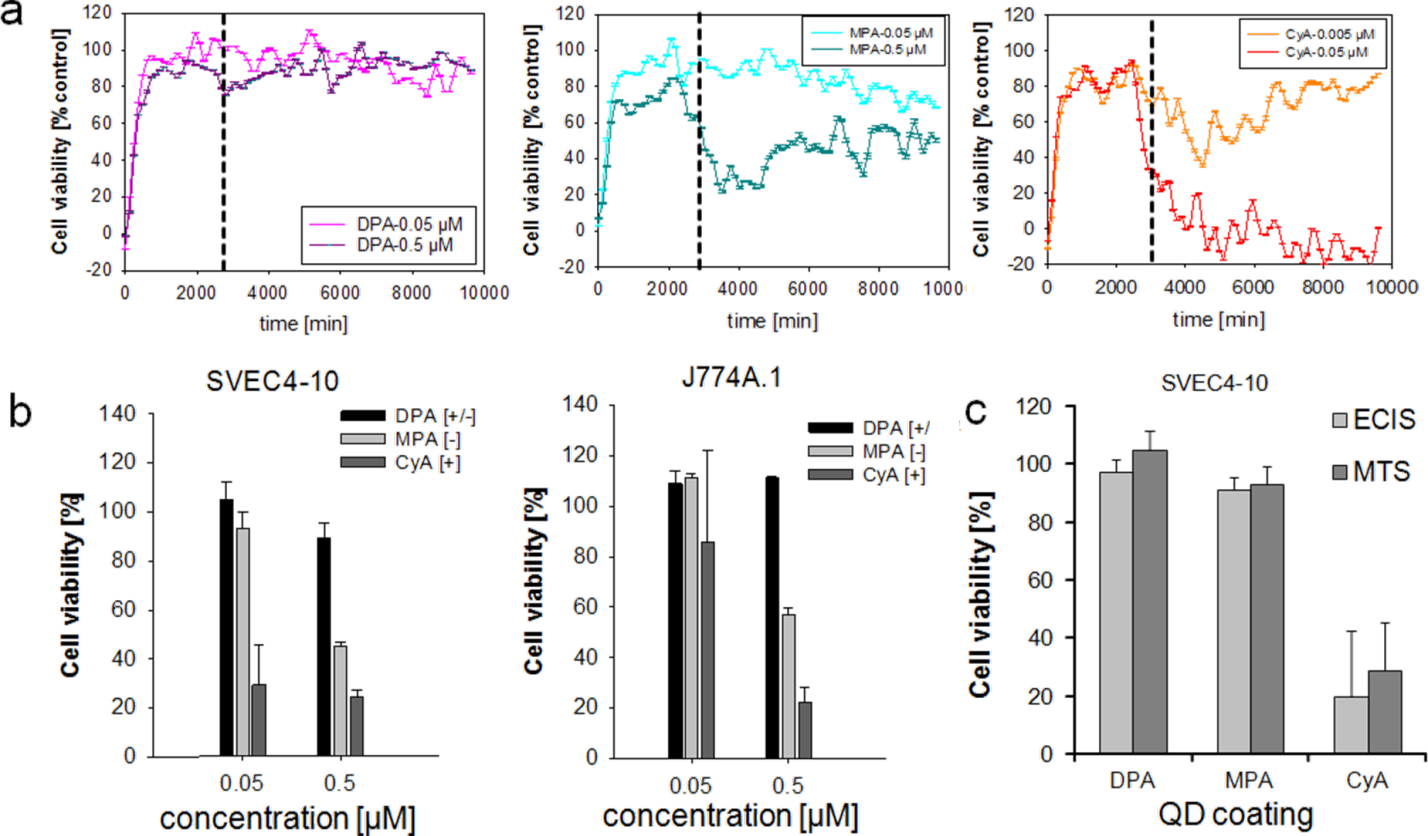
Comparative impact of quantum dots (QDs) with different surface coatings on cells measured after 24 h of incubation. (a) SVEC4-10 cells were treated with different QDs. Cell viability was determined by impedance measurements (for details see text). The vertical dashed line indicates the time point of nanoparticle exposure. (b) Relative cellular viability detected by MTS assay of endothelial cells (SVEC4-10) and of macrophages (J774A.1). (c) Direct comparison of both methods described in (a) and (b). MTS data were normalized to control values (no particle exposure, native), which were set to 100%. DPA: D-penicillamine, MPA: 3-mercaptopropionic acid, CyA: cysteamine.

The evaluation of the most suitable number of ZnS monolayers around the CdSe core of QDs showed that the lowest impact on cell metabolism (cellular dehydrogenase activities) was present when QDs were coated with two monolayers (Figure S1, Supporting Information File 1). Impedance measurements (electric cell–substrate impedance sensing, ECIS) revealed a distinct decrease in cell viability in the presence of cysteamine (CyA)-coated nanoparticles after incubation of endothelial cells with QDs with different surface coatings (Figure 2a). This decrease was concentration dependent. This effect was less pronounced for the 3-mercaptopropionic acid (MPA)-coated nanoparticles and nearly absent for the D-penicillamine (DPA)-coated QDs. These effects most certainly depend upon the positive charge of CyA, which resulted in an electrostatic attraction to the negatively charged cell membranes. Beyond impedance measurements, the MTS assay with endothelial cells (SVEC4-10) and macrophages (J774A.1) revealed comparable effects but the strongest decrease in cell viability was detected for the CyA coating (Figure 2b). A direct comparison of both methods confirmed the findings described above (Figure 2c). In particular, the biocompatibility of CdSe QDs depended on the surface functionalization and had the highest impact on the cell viability for the positively charged QDs. These findings confirmed the results of previous studies and further reinforce the fact that the cytotoxicity of positively charged nanoparticles is mainly due to impairment of cellular membranes.

Investigations of Hoshino et al. agree with our findings, a good biocompatibility was obtained with QDs-OH bearing a negative charge of −48 mV [48]. In contrast, positively charged QD-NH_2_ (+40 mV) as well as strongly negatively charged QD-COOH (−58 mV) led to DNA damages [48]. Therefore, we conclude that an intensive charge influences the cell viability. These effects on cell survival are probably attributed to the subcellular localization in nuclear and cytoplasmic compartments of cationic QDs [49]. Our comparison of different QDs reinforced the finding that positively charged ones have, in general, a lower biocompatibility compared to neutral or negatively charged QDs.

### Size affects the cellular metabolism

**Figure 3:**
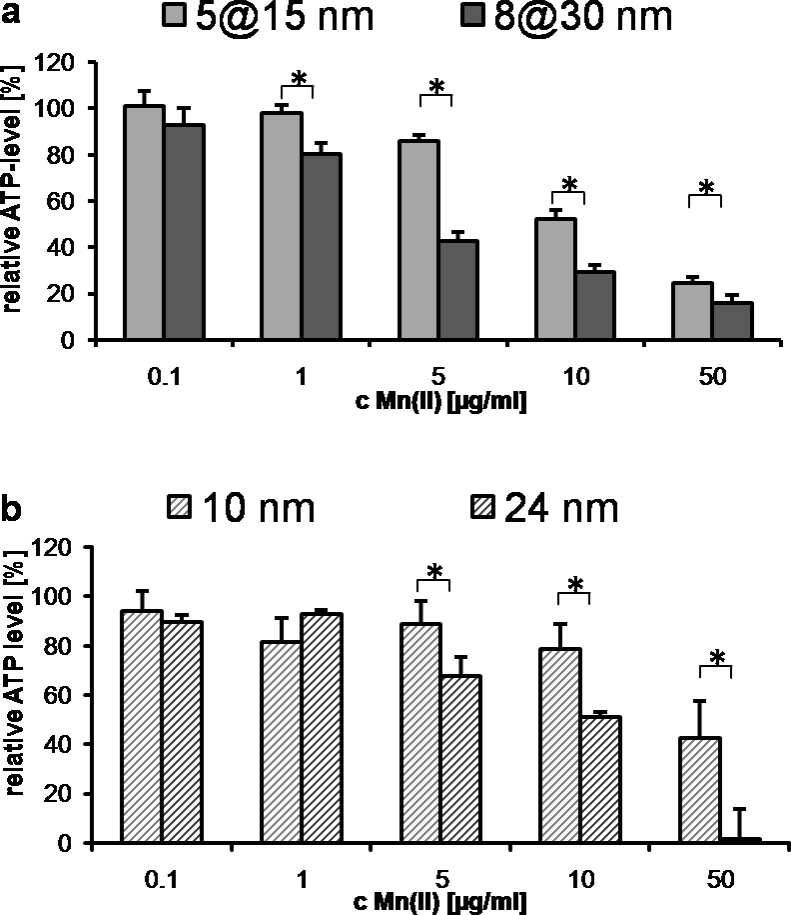
Size effects of the different manganese oxide nanoparticle formulations on the cellular ATP levels of endothelial cells, reflecting activity of cell metabolism. (a) Cells were treated with Au@MnO Janus particles. Relative ATP level was measured by the ATPLite assay after cells were incubated for 24 h with 0.1–50 μg/mL Mn(II). (b) Cells were treated with spherical MnO nanoparticles. Relative ATP level of HMEC-1 after incubation for 24 h with 0.1–50 μg/mL Mn(II). * *p* < 0.05

In general, distinct effects on cellular metabolism and uptake were detectable as a function of the nanoparticle size (5@15 nm vs 8@30 nm, Au@MnO) (Figure 3). Larger particles led to a more pronounced decrease of the cellular ATP levels than smaller ones. Nevertheless, these findings appear not to be specific for the asymmetric structure of the Au@MnO particles, as spherical nanoparticles exhibit a similar behavior (Figure 3b, 10 nm vs 24 nm MnO domain). This aspect has been corroborated by other studies on spherical nanoparticles demonstrating that larger nanoparticles exhibit a higher cytotoxic potential than smaller ones [50–51]. Therefore, the size-dependency rules already known for spherical nanoparticles also apply for particles with Janus features.

### Nanoparticle internalization depends on surface charge

**Figure 4:**
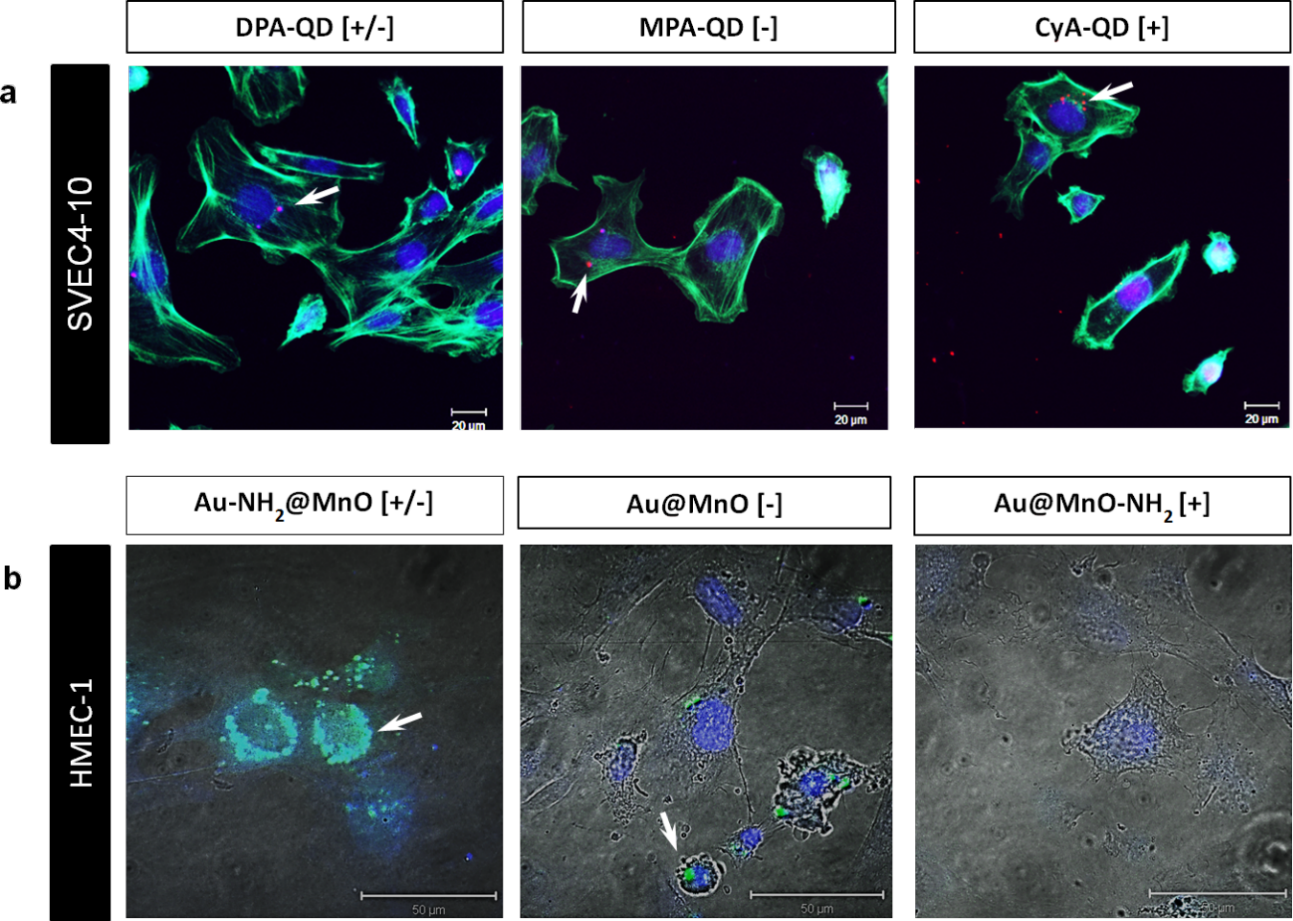
Internalization of different nanoparticles by endothelial cells depends mainly on the surface charge. Microscopical analysis of nanoparticle uptake after 24 h of incubation: (a) SVEC4-10 after treatment with quantum dots (QDs). The QDs are indicated in red (red fluorescence), the cell membrane in green and the nucleus in blue. Scale bar: 20 µm. (b) HMEC-1 after treatment with the Au@MnO nanoparticles indicated in green. The nucleus was stained with Hoechst (in blue). White arrows point to internalized nanoparticles. Scale bars indicate 50 µm.

The extent of internalization of the different QDs and the Au@MnO particles was demonstrated by confocal laser scanning microscopy (Figure 4). After 24 h of incubation, all QD formulations were visible as red dots inside the cells (Figure 4a). Interestingly, the positively charged variant showed the highest uptake intensity (Figure 4a CyA). Neutral Au-NH_2_@MnO particles were taken up to a higher extent than non-functionalized or particles that were functionalized at the MnO domain (Figure 4b). These findings are in agreement with other studies and explain the high cytotoxicity of the positively charged CyA-coated QDs. Positively charged gold nanoparticles were taken up by HepG2 cells to a higher extent than negatively charged ones. The internalization of these nanoparticles was similar in phagocytotically active cells, during which the nanoparticle charge did not play an important role [52]. Indeed, iron oxide nanoparticles with a positively charged surface coating showed a higher uptake level but also a lower stability compared to negative and neutral particles [53]. The stronger agglomeration behavior of positively or neutrally charged nanoparticles was also detectable in our studies and probably led to a higher uptake rate. Interestingly, Chen et al. observed a charge-dependent localization of mesoporous silica nanoparticles with positively charged particles in the cytosol and negatively charged ones in acidic endosomes [54]. Not only the surface coating but also the nanoparticle material and the cell type-specific internalization pathways seem to determine the uptake and trafficking of nanoparticles inside the cells.

### Co-localization of nanoparticles within cell organelles

**Figure 5:**
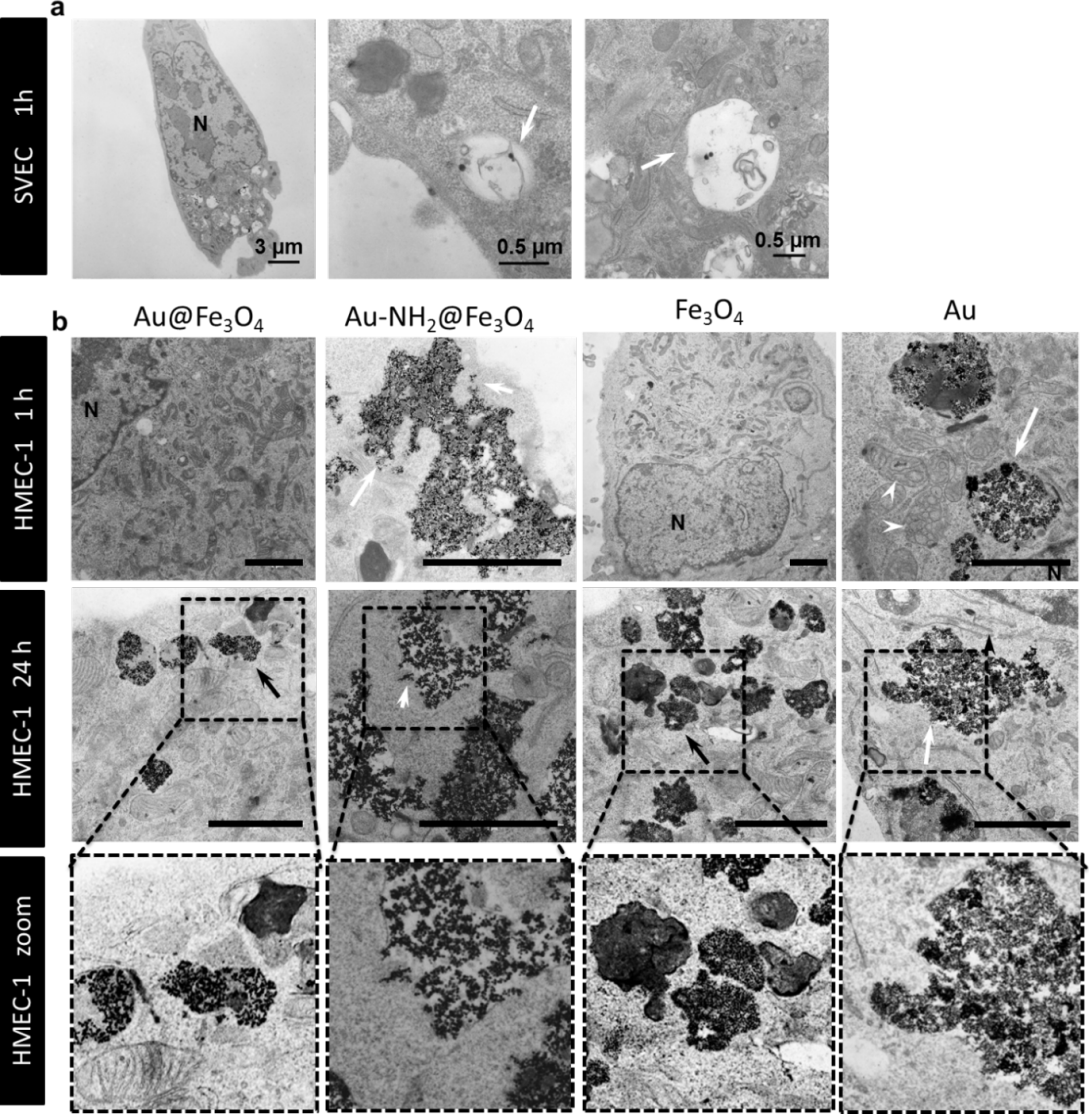
Transmission electron microscopy (TEM) images of different endothelial cells determined after 1 h and 24 h of nanoparticle incubation. (a) SVEC4-10 cells were treated with 30 µg/mL of PEGylated neutral charged gold spheres. They were localized in vacuoles (white arrow). (b) HMEC-1 cells were treated with 5 μg/mL Fe (II)/(III) of Au@Fe_3_O_4_, Au@Fe_3_O_4_-NH_2_ and Fe_3_O_4_ or with 5µg/mL of spherical gold (Au) nanoparticles (Nanopartz). All nanoparticles are localized in lysosomes and endosomes. Au@Fe_3_O_4_ and Fe_3_O_4_ are stored in small and clear delineated endosomes and secondary lysosomes (black arrows). White arrows point to damaged intracellular membranes. White arrow heads point to affected mitochondria. Scale bars in (b) indicate 2 µm. N = nucleus.

To analyze the localization of different nanoparticle formulations inside endothelial cells, transmission electron microscopy (TEM) investigations were performed. Spherical CTAB-modified gold nanoparticles with a size of 40 nm were localized in vacuoles after 1 h of incubation (Figure 5a). After a 1 h treatment of cells only, Au-NH_2_@Fe_3_O_4_ (20 nm) and spherical Au (4 nm) nanoparticles were shown to be internalized into endosomes (Figure 5b). Already after 1 h, damage in the endosomal and lysosomal membranes was observed (Figure 5b, white arrow). In contrast, non-functionalized Au@Fe_3_O_4_ and Fe_3_O_4_ particles were not visible inside the cells after 1 h of exposure (Figure 5b). TEM examinations after 24 h revealed localization of all variants (Au@Fe_3_O_4_, Au-NH_2_@Fe_3_O_4_, Fe_3_O_4_ and Au) in endosomes and a final deposition in lysosomes (Figure 5b). The good biocompatibility of the bare Au@Fe_3_O_4_ and Fe_3_O_4_ nanoparticles can be explained by the presence of small and clear delineated endosomes and secondary lysosomes (Figure 5b, black arrows). Au-NH_2_@Fe_3_O_4_ and Au were stored in large endosomes and lysosomes scattered over the entire cytoplasm. Furthermore, disrupted endosomal as well as lysosomal membranes caused by these nanoparticle types led to the release of the nanoparticles into the cytoplasm and thus affecting the mitochondria in the immediate vicinity (Figure 5b, white arrow heads). Moreover, it is possible that the cells get rid of these nanoparticles leading to the loss in cell viability.

In summary, the size of a nanoparticle defines its intracellular localization. Nanoparticles with a size of 40 nm are localized in vacuoles and 4 to 20 nm sized ones in endosomes and lysosomes. A smaller size (4 nm) as well as coupling of polar groups (e.g., NH_2_) accelerate the uptake and result in the loss of cell viability.

### Uptake mechanisms

**Figure 6:**
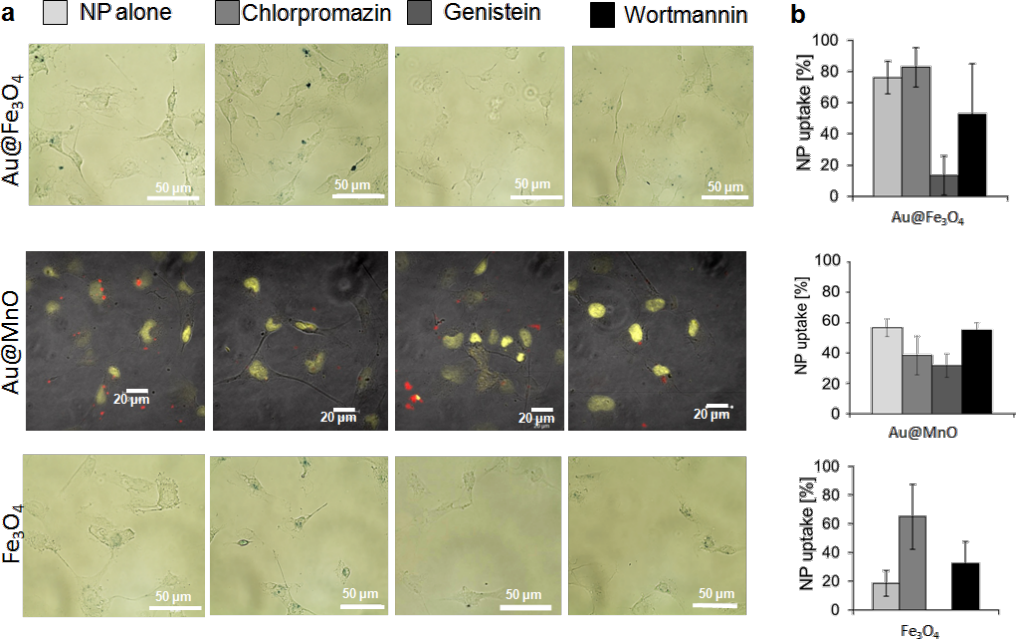
Microscopy images of endothelial cells and semi quantitative analysis of nanoparticle uptake to determine the endocytosis pathways of different metal oxide nanoparticles. HMEC-1 cells were treated with 5 µg/mL metal oxide Janus (Au@Fe_3_O_4_ and Au@MnO) or spherical (Fe_3_O_4_) nanoparticles after incubation with three different endocytosis inhibitors: chlorpromazine (clathrin), genistein (caveolae) or wortmannin (macropinocytosis) (for details see text). (a) Microscopical analysis of the cells and (b) semi quantitative analysis of the uptake (for further details see text).

In order to assess the mechanisms of endocytosis that are used for the internalization of different nanoparticle formulations, we used different inhibitors to block the best-known internalization routes: clathrin-mediated, caveolae-mediated and macropinocytosis [46]. Microscopy data and the semi-quantitative analysis of the nanoparticle uptake behavior revealed a caveolae-dependent internalization of Au@Fe_3_O_4_, Au@MnO and Fe_3_O_4_ particles (Figure 6). Caveolae-mediated uptake was blocked by the use of genistein, which was effectively demonstrated for anionic polystyrene nanoparticles in Hela cells [55]. Contrarily, Fernando et al. observed no changes for the internalization route of polymer nanoparticles by macrophages after the treatment with genistein [56]. Interestingly, the application of chlorpromazine, selectively affecting clathrin-mediated endocytosis [57–58], led to an increased accumulation of Au@ Fe_3_O_4_ and Fe_3_O_4_ nanoparticles in HMEC-1 (Figure 6a and Figure 6c).

**Figure 7:**
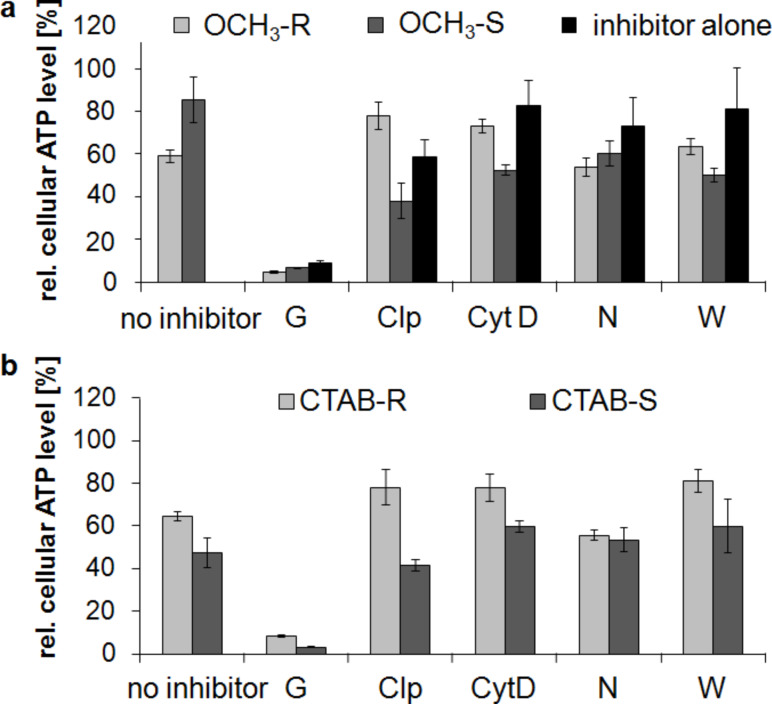
Impact of gold nanoparticles on cellular ATP levels of endothelial cells after the use of different endocytosis inhibitors. (a) SVEC4-10 cells were treated with OCH_3_ functionalized gold nanoparticles and different endocytosis inhibitors (R = rods, S = spheres). (b) SVEC4-10 cells were treated with CTAB modified gold nanoparticles and endocytosis inhibitors (R = rods, S = spheres). ATP data were normalized to control values (no particle exposure, native), which were set to 100%. Endocytosis inhibitors: G = genistein, Clp = chlorpromazine, CytD = cytochrome D, N = nocodazol, W = wortmannin.

After incubation of SVEC4-10 cells with OCH_3_-functionalized gold rods, the cellular ATP levels decreased to 60 to 80% (compared to untreated controls) after treatment with chlorpromazine and cytochalasin D, which indicates a clathrin- and macropinocytotis-mediated uptake of this nanoparticle type (Figure 7a, OCH_3_-R). The presence of the endocytosis inhibitors nocodazol and wortmannin had no significant effect on the cell metabolism. No changes of cellular ATP levels occurred after the use of the different inhibitors in the presence of OCH_3_-functionalized gold spheres. This observation is attributed to the low cytotoxic effects of the spheres (Figure 7a, OCH_3_-S). With the exception of genistein, the used inhibitors alone had no effect on the ATP levels (Figure 7a, inhibitor alone). Genistein as a blocking agent for caveolae-mediated endocytosis is known to strongly reduce cellular ATP levels independent of the used nanoparticle type due to the inhibition of ATP utilizing enzymes [59]. A strong cytotoxicity of genistein per se on endothelial cells could be excluded by the intact cell morphology shown in Figure S2 (Supporting Information File 1). The incubation of cells with CTAB-modified gold rods and spheres showed a similar uptake behavior compared to the OCH_3_-functionalized gold colloids, indicating a clathrin- and macropinocytosis-dependent mechanism (Figure 7b). Our analysis of uptake mechanisms of different gold nanoparticle formulations confirms literature data, in which a clathrin- and macropinocytosis-dependent uptake of anionic and cationic nanoparticles was commonly reported [43,60].

Some studies summarized that it is difficult to give general rules about a predicted internalization way depending on the type of nanoparticle [61]. Interestingly, in literature the internalization of nanoparticles seems to be a cell-type-specific process [61–63]. In this context, the mouse macrophages cell line J774A.1 used macropinocytosis and clathrin-mediated endocytosis for the uptake of 40 nm sized polystyrene nanoparticles, depending on the lack of caveolin-1 expression in this cell line. In contrast, the human alveolar epithelial cell line A549 showed no nanoparticle uptake after the inhibition of caveolae- and clathrin-mediated endocytosis [62]. Such a cell-type-specific internalization was also demonstrated in our study due to the inhibition of several endocytosis mechanisms. We showed that the mouse derived cell line SVEC4-10 used clathrin- and macropinocytosis for the nanoparticle uptake. In contrast, the used human endothelial cell line HMEC-1 internalized different nanoparticle formulations via caveolae.

Another interesting aspect is that nanoparticle uptake also depends on the nanoparticle size, which was demonstrated by several studies. In the literature, clathrin-mediated endocytosis of 50 nm sized folate-decorated nanoparticles was demonstrated as compared to the caveolae-mediated uptake of 250 nm sized particles [64]. Our own comparative analysis of different nanoparticle formulations revealed size specific uptake effects. Neutrally coated PEG-OCH_3_ and negatively charged CTAB gold nanoparticles with a size of 40 nm enter the cells via clathrin-mediated endocytosis and phagocytosis, irrespective of the nanoparticle shape. The negatively charged metal oxide variants with a size of 16 to 20 nm were taken up by caveolae-mediated endocytosis, this holds true for Janus particles as well as for the spherical ones.

## Conclusion

This comparative investigation of different nanoparticle shapes, sizes and functionalization revealed five major rules for endothelial cells.

1) In general, an elongated shape of gold nanoparticle rods and gold@metal oxide Janus particles leads to a stronger reduction in cell metabolic activity. 2) Endothelial cells react sensitively towards positively charged surfaces, e.g., caused by the surfactants NH_2_ and CyA. 3) Internalization of nanoparticles is driven by a positive surface and a small nanoparticle size. Interestingly, in the case of Au-NH_2_@Fe_3_O_4_ and 4 nm sized Au nanoparticles, the rapid uptake into endosomes and lysosomes leads to disturbed membrane integrity and the release of the nanoparticles into the cytoplasm. 4) A comparison of smaller and larger Janus and spherical particles showed a size-dependent effect with a stronger impact on the cellular ATP level of the larger ones (5@15 vs 8@30 nm and 10 vs 24 nm). 5) Endocytosis is probably a size-dependent process with caveolae-mediated uptake of nanoparticles around 20 nm and clathrin- or macropincocytosis-mediated internalization of nanoparticles greater than 40 nm.

A detailed understanding of these processes in endothelial cells is essential in order to design nanomaterials with specified characteristics for a defined nanotechnological application in vivo.

## Experimental

### Synthesis and characterization of the different nanoparticle formulations

To investigate the effects of the shape, functionalization, size and composition of various nanoparticles on endothelial cells we used gold colloids (GNP), asymmetric gold@metal oxide Janus particles (Au@MnO or Au@Fe_3_O_4_), spherical metal oxides (MnO or Fe_3_O_4_) and quantum dots (QDs). 1) Gold nanoparticles (GNP): The gold colloids with a core size of approximately 40 nm in diameter were synthesized according to Rosman et al. [45] and were spherically or rod-like shaped. GNP without surface functionalization were transferred to 0.1 M cetyltrimetylammonium bromide (CTAB) in water. Furthermore, we investigated the effects of GNP that were linked to thiol-terminated polyethylene glycol (SH-PEG, MW of PEG: 5 kDa) with different reactive groups: cationic (-NH_2_), anionic (-COOH) or neutral (-OCH_3_). 2) Asymmetric Au@metal oxide nanoparticles have an elongated shape and consist of a gold domain with a core size of 3.5 nm or 8 nm. The gold domain was synthesized according to Peng et al. [65] and functionalized with 1-octadecanethiole baring a hydrophobic surface. The linkage of metal oxides (Fe_3_O_4_ or MnO) of 15 nm or 30 nm in diameter to the gold domain was prepared by a seed-mediated chemical protocol [28,66]. 3) Spherical colloids of Fe_3_O_4_ (16 nm) and MnO (24 nm) were synthesized by thermal decomposition of iron(III) oleate or manganese(II) oleate in 1-octadecene described previously [67–68]. The metal oxide components of 2) and 3) were coated with a silica-FITC-PEG-shell presenting a hydrophilic surface [69]. For the NH_2_-functionalization 3-aminopropyltriethoxysilane was used for the second condensation reaction. As a control, we purchased spherical gold nanoparticles with a core size of 4 nm and an octadecanethiole shell from Nanopartz (Nanopartz^TM^ Inc., USA). 4) Semiconductor quantum dots (QDs): The synthesis of spherical CdSe core quantum dots followed a prescription from Mahler et al. [70] and the ZnS capping procedure (with 0, 2, 4, 6 ZnS monolayers (ML), size: 2.8, 4.0, 5.4, 6.7 nm) was peformed according to Breus et al. [71]. For further functionalization three different ligands for CdSe/4 ZnS core–shell QDs (*d* = 5.4 nm) were selected: 3-mercaptopropionic acid/MPA (COOH), cysteamine/CyA (NH_2_), D-penicillamine/DPA (NH_2_/COOH). These coatings represent neutral, positive or negative charge and provide the solubility in water. The calculated amount of a ligand (57 mg CyA, 44 µL MPA, 75 mg DPA) was dissolved in 20 mL of 2-propanol (for CyA and DPA) or 20 mL 1:1 methanol/dioxane mixture (for MPA). The pH was adjusted to 11–12 with tetramethylammonium hydroxide pentahydrate (TMAHP) in case of MPA and DPA, and the mixture was heated up to 70 °C upon fast stirring under argon flow. An amount of 0.2 mL of 100 μM CdSe/ZnS QD stock solution in chloroform was added to the refluxing solution and kept under stirring for 10–15 min before cooling down to RT. Ethyl acetate was added to precipitate the ligand-exchanged QDs. The sample was centrifuged for 40 min at 4000 rpm and the resulting pellet was redissolved in 1 mL of distilled water.

Prior to the measurements, nanoparticles were sonicated (Sonorex RK 52 H, Bandlein, Germany) in deionized water. Afterwards the NPs were suspended in complete culture medium with increasing concentrations according to the experiment and the cell line.

### Cell culture

The mouse endothelial (SVEC4-10) cell line was obtained from American Type Culture Collection (LGC Standards GmbH, Germany). Cells were grown in Dulbecco´s modified eagle´s medium (DMEM with Glutamax; Invitrogen, Germany) supplemented with 10% heat-inactivated fetal calf serum (FCS, Invitrogen, Germany). The immortalized human micro vascular endothelial cells (HMEC-1; Centers for Disease Control and Prevention, USA) were cultured in Gibco^®^ MCDB 131 medium supplemented with 10% (v/v) FCS, 1% (v/v) GlutaMAX^TM^ I (100×; Life Technologies GmbH, Germany), 1 µg/mL hydrocholesterol (Sigma-Aldrich Chemie GmbH, Germany), and 10 ng/mL epidermal growth factor (Life Technologies GmbH, Germany). Mouse macrophages (J774A.1) used as control cell line were obtained from American Type Culture Collection (LGC Standards GmbH, Germany) and cultured under the same conditions as described above for the SVEC4-10 cells. All used cell lines were cultured at 37 °C in a 5% CO_2_ humidified environment. For experimentation, all cells were plated onto a plastic matrix at a density of 1.2 × 10^4^ cells/cm^2^ (endothelial cells HMEC-1), 4.4 × 10^3^ cells/cm^2^ (endothelial cells SVEC4-10) or 8.8 × 10^3^ cells/cm^2^ (macrophages J774A.1). They were allowed to grow for 24 h before nanoparticle exposure. The cells were negative for mycoplasma as routinely determined via PCR.

### Cell viability plate assays (MTS and ATP)

To determine the effects of different nanoparticles on cell metabolism, we used a colorimetric (MTS: 3-(4,5-dimethylthiazol-2-yl)-5-(3-carboxymethoxyphenyl)-2-(4-sulfophenyl)-2*H*-tetrazolium, inner salt; Aqueous One Solution Cell Proliferation Assay, Promega, Germany) and a luminescence (ATPLite assay, PerkinElmer, Germany) based cytotoxicity assay. In this context, cells were seeded into the respective clear or white 96-well plate (Greiner, Germany) depending on the assay read-out and grown overnight (37 °C, 5% CO_2_). Cells were exposed to increasing concentrations of nanoparticles for different incubation times up to 72 h. Afterwards, cells were washed three times with HBSS (Hanks´ BSS; PAA Laboratories GmbH, Germany) to remove non-internalized nanoparticles. Assays were carried out according to the instructions of the manufacturer. Reduction of MTS into a soluble formazan product was assessed by measuring the absorbance at 492 nm in a microtiter plate reader (Tecan, Germany) after a 4 h incubation period at 37 °C. The production of light was measured with a luminescence plate reader (BMG LABATECH GmbH, Germany). Relative cellular dehydrogenase and ATP levels were expressed as relative values to the untreated control.

### Measuring cell viability through impedance

Endothelial cells (SVEC4-10) were harvested from culture dishes and seeded in an 8-well electrode array at the density of 60.000 cells/well (equates confluency) at 37 °C in a humidified 5% CO_2_ environment. Exchange of culture medium with medium containing CdSe/4ZnS core-shell QDs (with CyA, MPA, DPA) was carried out 24 h after seeding. All these experimental procedures occured during the electric cell–substrate impedance sensing (ECIS) recording and the 8-well electrode array was placed at 37 °C in the humidified 5% CO_2_ environment (incubator CO2Cell, Germany). Adherent cells spread on the surface of planar gold-film electrodes and increase the impedance of these electrodes [72]. A custom-built ECIS system was employed, consisting of a lock-in amplifier (SR830, SRS, Inc., CA) with an internal oscillator, a multiplexer with analogue switches for automatic, consecutive addressing of individual wells on the electrode array, and a PC for experiment control and data storage. The ECIS electrode arrays (type 8W1E) purchased from Applied Biophysics (Troy, USA) consisted of eight separate wells, each holding one gold microelectrode of 250 μm diameter and a large (7–46 mm^2^) counter electrode. In our ECIS setup, a 1 V AC signal was applied to the system through a 1 MΩ series resistor, and the in-phase and out-of-phase voltages across the electrodes was recorded at 4 kHz at a sampling rate of 1 Hz. For micromotion recordings, the in-phase voltage, which is directly proportional to the real part of the complex impedance, was used for further analysis as it provides the most sensitive readout. As suggested by Giaever and co-workers [73], micromotion data, which are essentially a time series of resistance fluctuations, were subjected to Fourier transformation after subtracting a linear trend. Raw data of micromotion measurements (three independent experiments) were transferred to Excel and were assessed for cell viability. The micromotion values were normalized by the controls (medium only) and expressed as percent viability.

### Uptake analysis via fluorescence microscopy

To analyze the uptake of fluorescent nanoparticles, endothelial cells were treated with the corresponding nanoparticle formulations. After washing to remove non-internalized particles, cells were fixed for 10 min in 4% (v/v) formaldehyde at 4 °C. The green fluorescent nanoparticles (QDs and Au@MnO) were detected via fluorescence microscopy (CLSM) (LSM 510 Meta, Carl Zeiss Micro Imaging GmbH, Germany) and the cell nuclei were stained with Hoechst 33258 (Applichem, Germany) in blue. Further on, the glycocalyx was stained in red with lectin WGA-AlexaFluor633 (Invitrogen GmbH, Germany).

### Intracellular localisation of nanoparticles — electron microscopy

For TEM analysis cells were cultured in 24-well plates as described above until they reached 90% confluency. Endothelial cells were treated with different nanoparticle formulations for 1 and 24 h. Subsequently, cells were fixed with 2% (v/v) glutaraldehyde solution (EM grade in 0.1 M cacodylate buffer, pH 7.4) containing 5% sucrose for 30 min at 20 °C. After washing cells with cacodylate buffer (0.1 M, pH 7.4, 6.8% sucrose), they were post-fixed with a freshly prepared mixture of one volume 2% OsO_4_ (dissolved in distilled water) and one volume of 3% K_4_[Fe(CN)_6_] in 0.2 M cacodylate buffer (pH 7.4) at 4 °C for 2 h. Cells were rinsed with 0.1 M cacodylate buffer until the solution remained clear, dehydrated in graded series of ethanol and embedded in Epon 812 via hydroxypropyl methacrylate as intermedium. The Epon sheets with the cell layers were dried in an oven at 60 °C for six days. A low-angle diamond knife (Diatome, Biel, Switzerland) in a Leica Ultracut S ultramicrotome was used to make ultrathin sections and then staining with freshly prepared uranyl acetate and lead citrate was performed. The sections were evaluated using a transmission electron microscope EM 902A (Zeiss, Germany) at an accelerating voltage 80 kV.

### Incubation of cells in the presence of selected drugs inhibiting specific metabolic pathways

Effects of GNP and asymmetric Janus particles on total cellular ATP level and uptake in the presence of selective drugs blocking specific metabolic pathways associated with endocytotic processes were assessed. SVEC4-10 cells were pre-incubated with 5 µg/mL cytochalasin D (inhibitor of actin polymerization, indicator of caveolae and macropinocytosis) [74], 100 nM wortmannin (inhibitor of PI3 kinase, indicator of macropinocytosis) [75], 5 µg/mL nocodazol (promotes microtubule depolimerization) [74,76], 10 µg/mL chlorpromazine and 200 µM genistein (inhibitor of tyrosine kinase, caveolae mediated) [57] for 1 h. For the treatment of HMEC-1 43 ng/mL wortmannin, 100 ng/mL chlorpromazine and 40 µg/mL genistein were used. Afterwards, CTAB-modified GNP (spheres or rods), or metal oxide containing Janus nanoparticles were added and incubated for additional one (SVEC4-10) or three (HMEC-1) hours. Then, the total cellular ATP levels or the changes in nanoparticle uptake were assessed microscopically as described above. The effects related to the presence of GNP alone or the respective drugs were monitored via appropriate controls without nanoparticles and without inhibitors.

### Semi-quantitative analysis of the nanoparticle uptake

After treatment of HMEC-1 with endocytosis inhibitors and nanoparticles, a semi quantitative determination of the amount of internalized nanoparticles was done by microscopic analysis of the cells. Three different field-of-views, with a surface area of 74 mm^2^ each, were randomly selected and the number of all visible cells was counted. Afterwards, fluorescence (FITC) or bright-field microscopy (Prussian blue) was completed to measure the presence of cells loaded with nanoparticles. The uptake rate was normalized as the percentage of cells with nanoparticles.

## Supporting Information

File 1 Additional figures.
