# Supplementary material for: Comparative evaluation of the impact on endothelial cells induced by different nanoparticle structures and functionalization
Source: Beilstein J Nanotechnol. 2015 Jan 27;6:300–12. doi: 10.3762/bjnano.6.28 (PMC4362490; doi:10.3762/bjnano.6.28)
Supplement: File 1 — Additional figures. [file Beilstein_J_Nanotechnol-06-300-s001.pdf]

Supporting Information

for

Comparative evaluation of the impact on endothelial cells induced by different nanoparticle structures and functionalization

Lisa Landgraf*[§1], Ines Müller[1], Peter Ernst[1], Miriam Schäfer[1], Christina Rosman[2], Isabel Schick[3], Oskar Köhler[3], Hartmut Oehring[4], Vladimir V. Breus[5], Thomas Basché[5], Carsten Sönnichsen[2], Wolfgang Tremel[3] and Ingrid Hilger*[§1]

Address: [1]Institut für Diagnostische und Interventionelle Radiologie des Klinikums der Friedrich-Schiller-Universität Jena, Bachstraße 18, D-07740 Jena, Germany; [2]Institut für Physikalische Chemie, Johannes Gutenberg Universität Mainz, Duesbergweg 10-14, D-55128 Mainz, Germany; [3]Johannes Gutenberg-Universität, Institut für Anorganische Chemie und Analytische Chemie, Duesbergweg 10-14, D-55128 Mainz, Germany; [4]Institut für Anatomie II, Universitätsklinikum Jena, Teichgraben 7, D-07743 Jena, Germany and [5]Institut für Physikalische Chemie, Johannes Gutenberg Universität Mainz, Duesbergweg 10-14, D-55128 Mainz, Germany

Email: Lisa Landgraf* - Lisa.Landgraf@med.uni-jena.de;
Ingrid Hilger* - ingrid.hilger@med.uni-jena.de

* Corresponding author

[§]Institut für Diagnostische und Interventionelle Radiologie des Klinikums der Friedrich-Schiller-Universität Jena, Forschungszentrum Lobeda, Erlanger Allee 111, D-07747 Jena, Germany. Phone: 0049-(0)3641-9325921, Fax: 0049-3641-9325922

Additional figures

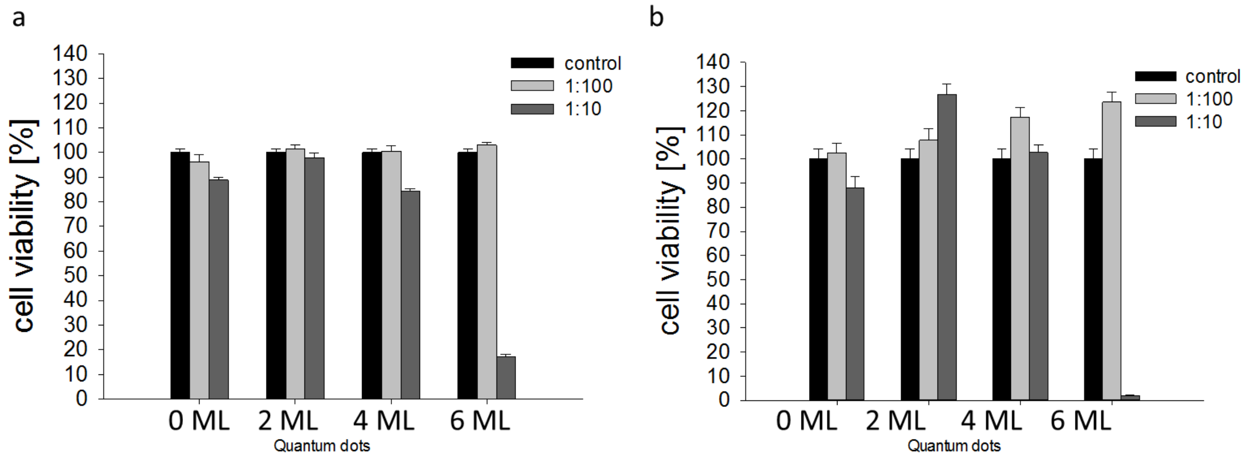

Figure S1: Cell viability of endothelial SVEC4-10 cells after exposure to different numbers of monolayers (0 to 6 ML) QDs after 24 h (a) and 72 h (b) of incubation as measured by the MTS assay.

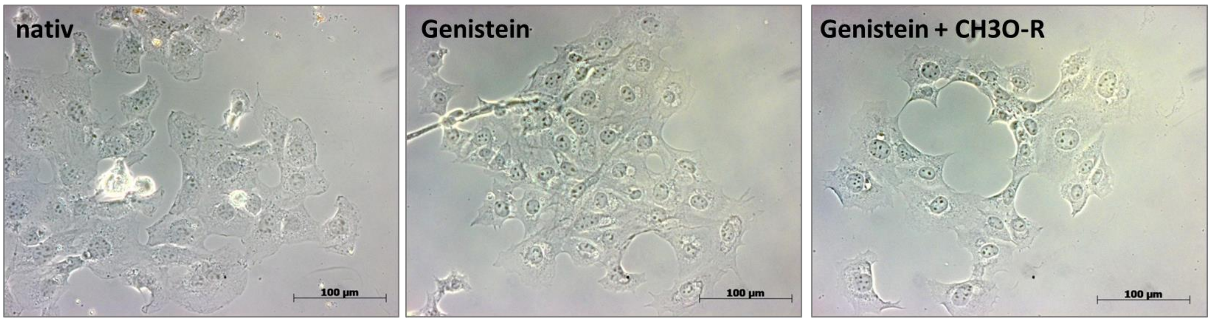

Figure S2: Microscopical analaysis of SVEC4-10 cells after treatment with endocytosis inhibitor genistein and CH_3O functionalized gold nanoparticles. Scale bars indicate 100 µm.
